# Barriers to clinical cancer research participation: moving from inclusion to engagement when considering European migrants’ recruitment

**DOI:** 10.1186/s13063-025-09293-9

**Published:** 2025-12-23

**Authors:** N. Millet, M. Brzeska, B. Czyznikowska, G.-I. Postavaru, I. Silina, N. Darko, E. L. Moss

**Affiliations:** 1 College of Life Sciences, University of Leicester, Leicester, LE2 7LX UK; 2 University Hospitals of Leicester NHS Trust, Infirmary Square, Leicester, LE1 5WW UK; 3 Department of Medical Psychology, Amsterdam UMC, Location VUmc, Amsterdam, 1081 BT The Netherlands

**Keywords:** Research recruitment, Clinical cancer trials, Diversity, Engagement, European migrant population

## Abstract

**Background:**

Participation in clinical cancer research trials should diversely reflect the intersectionality characteristics of the general population for results to be representative and applicable. European migrant populations residing in the United Kingdom (UK) are a group whose participation in clinical research warrants further exploration from a community and clinical perspective. This study aimed to explore the barriers and facilitators of individuals who have migrated to the UK from an EU8 or EU2 (EU8/2) country to participating in clinical cancer research trials to update clinical and research agendas for optimising inclusive engagement strategies.

**Methods:**

Perspectives of migrant individuals and of clinical research staff were explored to identify barriers and opportunities for optimising engagement. Five focus groups with clinical research staff at four hospitals across the East Midlands and three online focus groups with individuals who had migrated to the UK from Poland, Latvia and Romania were conducted. Data was analysed using template analysis.

**Results:**

Twenty-two clinical research staff and 17 individuals from EU8/2 countries participated in the study. Three key themes and related subthemes were identified: (1) Ambivalence, misunderstanding and fear shape cancer research perceptions (1.1. a lack of familiarity with cancer research practices; 1.2. Cancer fear may hinder participation); (2) Structural barriers and gaps in cultural competency; and (3) Building trust through community engaged research (3.1.Co-researching with communities; 3.2. Incentivising and legitimising research).

**Discussion:**

Many migrant participants were unfamiliar with UK-based research practices, and it was suggested that fatalist attitudes towards a cancer diagnosis and mistrust of research generally created apprehension and defensiveness when hearing about clinical cancer research in migrant communities. Migrant individuals and staff endorsed research design strategies which engage community champions (including clinicians); narrate positive stories of cancer research participation; and consider language accessibility and comprehension as key elements of engagement-focused research design.

**Supplementary Information:**

The online version contains supplementary material available at 10.1186/s13063-025-09293-9.

## Background

Clinical trials represent research that is central to informing the efficacy and safety of healthcare interventions, which in turn contribute to guidelines that shape healthcare practices. Thus, participants within clinical trials should represent the diversity of service users whom the trial results aim to impact [1]. Globally, there is a growing focus on improving such representation [2, 3]. The need for such efforts was highlighted within SARS-COVID-19 trials, where underrepresentation and incorrect classification of ethnicity were reported despite the respiratory disease disproportionally affecting those from ethnic minority groups [4]. This also extends to studies aiming to represent a national population; for example, there is over-representation of samples from White ethnicity participants within international biobank data from cancer research studies, thus leading to calls for greater diversity within oncological research studies [5].

A recent systematic review found that tailoring recruitment strategies through engaging with underserved groups was the most used strategy to overcome barriers to diverse and inclusive recruitment [6]. Additionally, cultural competency and sensitivity training, establishing a diverse advisory panel and recruiting research staff from underrepresented groups, were key actions identified for promoting inclusion in clinical trials [7]. However, the recruitment of participants into clinical trials is already perceived to be burdensome for clinical research staff due to logistical regulatory challenges, time pressures and the psychological impact of this work [8, 9]. Therefore, the issue of representation needs to be addressed through sustained and systematic efforts to increase participant diversity and inclusion of various intersectionality characteristics (e.g. socioeconomic factors, geography and community or cultural factors) related to researched populations.

Identifying and addressing the potential barriers to participation [9] and the development of initiatives [10] and resources to improve participation may vary between underrepresented groups [11]. Such groups include those who have migrated to the UK from EU8 (Czech Republic, Estonia, Poland, Hungary, Latvia, Lithuania, Slovakia, Slovenia) or EU2 (Bulgaria and Romania) countries. Many migrants from EU8 and EU2 (EU 8/2) countries have the same level of access to the NHS as UK citizens under the EU settlement scheme [12]; however, the structure of health services, screening behaviours, and knowledge and trust of clinical research may differ substantially in the UK compared to migrants' countries of birth. According to the Wellcome Global Monitor 2018 [13], countries categorised as ‘Eastern European’ were amongst the group of regions that reported the least trust in doctors and nurses globally.

The 2021 National Census identified that 16.8% of the population resident in England and Wales were born outside of the UK, with approximately 5% of the population born in a European Union (EU) country [14]. In addition, Polish is now the most common non-UK nationality resident in England and Wales (738,000 individuals) and Romanian the third largest (384,000 individuals). Since ethnicity within the NHS is recorded using the Office for National Statistics (ONS) classification, many of the individuals from EU8/2 countries will self-identify as belonging to a *White: Other* ethnicity category, for example *White: Romanian* (1084), *White: Polish* (1025) or *White: Other Eastern European* (1038). Therefore, the participation of EU8/2 individuals in clinical trials is often difficult to dissect since they would be included within the overall *White ethnicity* classification and not as distinct ethnic groups.

Specific barriers for women who have migrated to the UK from an Eastern or Central European country have been explored with respect to cervical cancer screening where language and distrust of the healthcare system were identified as significant barriers to screening participation [15]. Findings from the NIHR’s INCLUDE project [16] indicate that those who encounter language barriers should be treated as an exemplar underserved group. To ensure equitable, not just equal care, interventions and clinical pathways need to be tailored to the specific needs of target populations, including language proficiency and care preferences [15]. To address this issue, our study aimed to (i) explore experienced and potential barriers to research participation for individuals who have migrated to the UK from EU8/2 countries, from both a participation and recruitment perspective, and (ii) identify recommendations for clinicians and researchers.

## Methods

### Study setting, research team and ethical approval

This study was conducted by researchers at the Leicester Cancer Research Centre at The University of Leicester (UoL) in collaboration with the Centre for Ethnic Health Research. The team includes researchers with lived experience of migration, including colleagues who have migrated to the UK from EU8/2 countries (e.g. Poland and Romania), working alongside clinicians (gynaecology, oncology) and social scientists (sociology, psychology). This interdisciplinarity and lived experience shaped culturally sensitive design choices, language and consent procedures, and recruitment approaches. It also strengthened interpretation through ongoing reflexive discussion of positionality, trust and power dynamics between researchers and participants. Several team members speak relevant European languages and have established community links, which supported communication and participant engagement. Throughout the study, reflexive discussions amongst team members with differing cultural and professional backgrounds helped contextualise participants’ accounts. Ethical approval for the study was gained through the Yorkshire & The Humber—Sheffield Research Ethics Committee (22/YH/0224) and Health Research Authority.

### Study design

A qualitative study was conducted to highlight clinical, research and community perspectives. The study was designed to explore experienced and potential barriers to clinical cancer trial participation from the perspective of individuals who have migrated to the UK (group 1) and to understand how these intersect with the experiences of those who design and recruit for clinical cancer research trials (group 2) to identify clinically relevant opportunities for optimising inclusive engagement strategies.

### Participants and recruitment

Given that migrant individuals from Poland, Romania and Latvia represent the largest EU8/2 populations within the East Midlands, participants were (i) members of the public who had migrated to the UK from Poland, Romania or Latvia (EU8/2 regions) and (ii) clinical research staff and healthcare professionals actively recruiting for research studies from hospitals in the East Midlands region.

Recruitment of migrant individuals was coordinated and led by a Centre for Ethnic Health Research (CfEHR) community engagement officer (BC), who is of Polish ethnicity. Migrant individuals who had personal experience of receiving a cancer diagnosis or who had experience of a close other receiving a cancer diagnosis and self-identified as having migrated to the UK from Poland, Latvia or Romania were purposefully recruited to represent the characteristics of the target population cohorts relevant to the study. Potential participants were reached through existing CfEHR outreach networks, WhatsApp groups and snowball sampling, facilitated by word-of-mouth, personal referrals and face-to-face engagement (by visiting existing groups within the target population cohorts) as well as online and remote platforms (such as email, contact with key community leaders and online community forums). To reach a broad range of participants, the outreach covered various geographical locations, including Leicestershire and Nottinghamshire. Some participants from the Polish cohort had previously taken part in public involvement activities through CfEHR. Participants from the Latvian and Romanian cohorts had not previously taken part in any CfEHR activities. Eligible participants were contacted at least 2 weeks in advance, and consent was obtained from each participant before data collection via email communication. Telephone conversations were held with some participants to explain the informed consent process and to provide support with submitting the form. A postal option for consent was also offered to accommodate varying accessibility needs; however, none of the participants opted for this method.

For the focus groups with clinical staff, we aimed to recruit a group of research staff working in various disciplines and departments in order to foster interdisciplinary learning with regard to optimising engagement practices. Clinical research staff and research-active healthcare professionals were recruited from four NHS hospitals in the East Midlands region. An open invitation including participant information was emailed through the participating hospitals’ research leadership teams who were known to the senior researcher (EM). The internal hospital leadership team representative assembled a group of research staff and coordinated their participation in the focus groups (including choosing a date, time and location that was suitable to their schedule). Informed consent was conducted in person by NM prior to conducting the in-person focus group sessions.

### Data collection

#### Focus groups with migrant individuals from EU8/2 countries

Three focus groups were conducted between November and December 2022, one each with participants who had migrated to the UK from Poland, Latvia or Romania. All focus groups were conducted by BC. The focus group involving Polish participants was conducted in the Polish language by BC, and the other two focus groups were conducted in English with Latvian and Romanian interpretation. BC has extensive experience in engaging with minority and migrant communities and has previously designed and conducted patient and public involvement and engagement activities, and facilitated multiple focus groups with diverse populations. This experience informed the culturally sensitive approach adopted throughout the study. Sociodemographic data (age, country of birth, religion, gender, languages spoken and number of living years in the UK) was collected prior to focus groups via a questionnaire that participants were asked to complete and return via email. Focus groups took place online using the Microsoft Teams platform (in line with participant preference) and followed a focus group topic guide. At the start of each focus group, the guidelines for interaction and feedback were explained to all participants. They were encouraged to raise their hand before speaking to avoid interrupting others. Participants were also reminded to take into account the presence of the interpreter. During sessions, some participants posted information in the chat in English. These messages were read aloud in both English and the relevant language for those who did not speak English. This approach helped manage responses from multiple participants and ensured that all contributions were accurately communicated and understood through the interpreter. Focus groups followed a semi-structured topic guide which explored both individual experiences (e.g. personal or family history of cancer, diagnosis, treatment, support and coping) and community perceptions (e.g. whether cancer is discussed in families or the community, common topics and cultural beliefs such as stigma or religious interpretations), alongside attitudes and experiences of participating in research. Prompts were used to explore cultural themes, gender differences, and to encourage participants to share both their personal experiences and their observations of broader community attitudes towards cancer. Focus groups were audio recorded and transcribed using Microsoft Teams software and checked for accuracy by BC.

#### Focus groups with clinical research staff

**Fig. 1:**
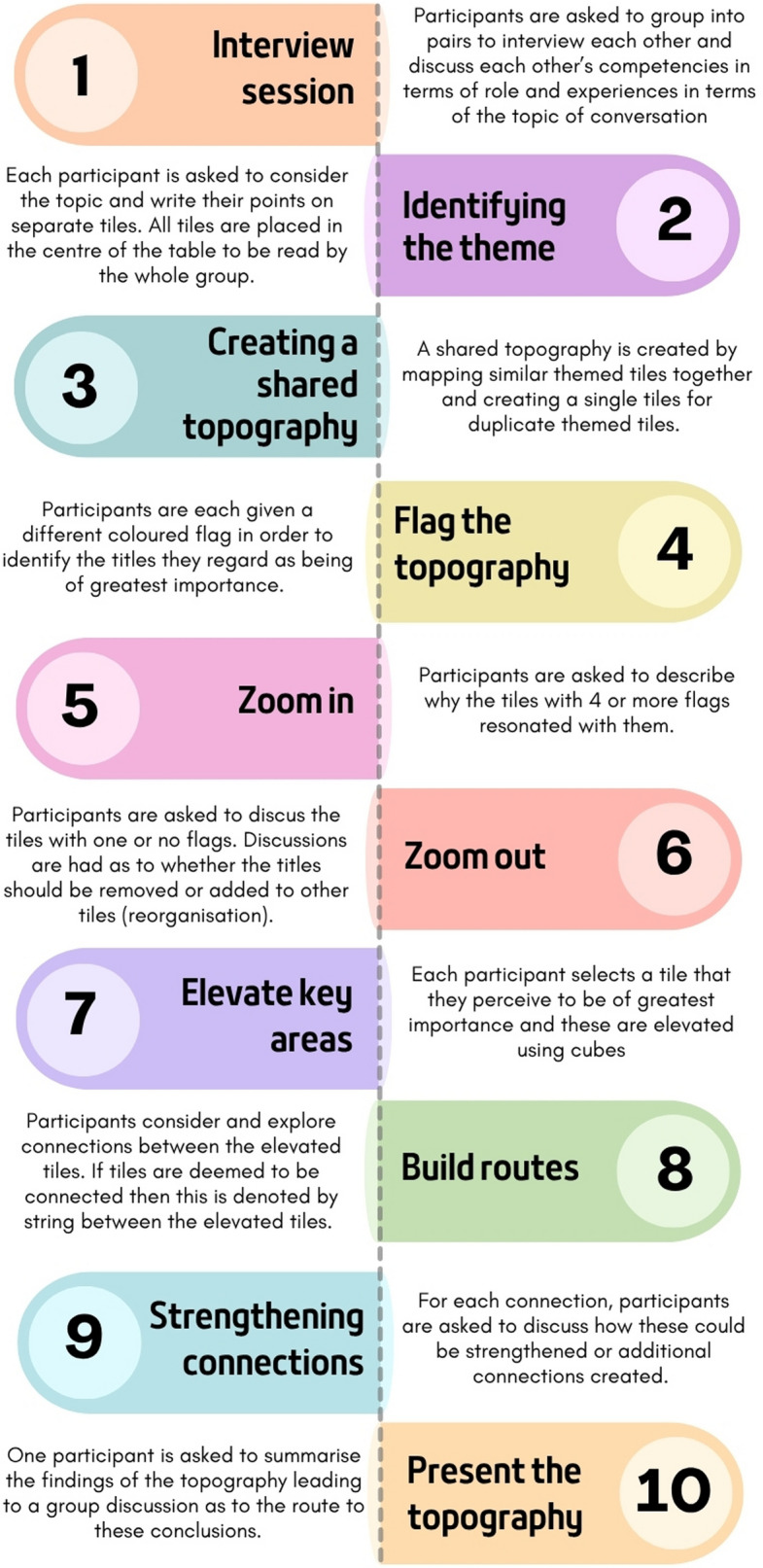
The 10 steps of the co-navigator kit approach for interdisciplinary problem solving

Five 2 hour, in-person focus groups were conducted at four hospitals across the East Midlands region in 2023. In order to facilitate discussion around potential barriers and opportunities for engaging with migrant populations in clinical trials, we chose to structure focus groups using the co-navigator kit approach [17]. This approach was originally developed by scholars to facilitate interdisciplinary collaborations with the goal of problem solving. Given that these focus groups consisted of clinical research staff with varying roles from differing clinical fields of study, this hands-on approach was well-suited to managing and elevating interdisciplinary voices. The focus groups were facilitated by NM who holds over 8 years of experience in collecting and analysing qualitative and focus group data, and who had previously received training in the co-navigator kit approach. The approach consists of 10 steps (Fig. 1) to facilitate discussions. It uses one overarching topic rather than an interview schedule. The topic that we formulated was ‘Effective engagement of Eastern European ethnicity individuals into clinical research trials’. In the first step, participants interviewed each other in pairs, asking about the other person’s competencies. Each person was then invited to relay their partner’s information to the rest of the group as a way to introduce them. Then, all participants were invited to consider how the formulated topic had meaning for each of them from their own clinical and/or research experience and to write this down on a moveable, erasable card (step 2). Each participant was then invited to explain what they had written which led to discussions amongst the group, particularly related to points of similar experiences. Then, the group used these discussions to start grouping their cards based on similarities and to refine and re-write cards where they overlapped (step 3). Steps 4 to 7 led to the elevation of the most salient cards as chosen by participants and summarising the main points of discussion. The output from these focus groups was photos of the written meanings in response to the topic and audio recordings of the workshops which were transcribed verbatim by NM. For the purpose of this study, only the audio recordings were used, meaning that both sets of focus group data (migrant individuals and clinical research staff) could be analysed together.

### Data analysis

A thematic ‘small q’ template analysis approach was followed [18, 19] whereby data was interrogated based on a priori groupings. A coding template was derived based on the aims of the research study which included four themes: attitudes towards cancer research; perceptions of cancer; barriers to effective engagement; and facilitators of effective engagement. The coding template was firstly deductively applied to the data set by NM and BC independently to enhance rigour. In doing so, the data transcripts were read through, and any underlying meaning associated with data segments was coded. These codes were then placed within the corresponding template theme. Where a code did not fit a pre-determined theme, an inductive approach to theme/sub-theme development was taken. Code allocation was discussed by BC and NM to resolve disagreements and to refine the codes further to highlight significant areas of importance and meanings related to the research question.

## Results

### Participants

#### Migrant individuals

Seventeen individuals from EU8/2 countries participated in a focus group which lasted on average 95 minutes. Relevant demographic factors (age, gender, country of birth, religion, languages spoken and number of years living in the UK) are detailed in Table 1 in a manner that protects participants’ identities. Participants recruited for the three focus groups broadly reflect the demographic characteristics of Romanian, Polish and Latvian migrants in the UK regarding age and language spoken, whilst women are over represented [20, 21]. The sample consisted mainly of working-age adults, with a range of backgrounds consistent with the wider migrant populations residing in the East Midlands. Some participants played active roles within their local communities, including participation in the voluntary sector and community organisations that provide welfare, advisory and support services to migrant networks.

**Table 1:** Sociodemographic characteristics of participants who self-identified as migrant individuals from Latvia, Poland and Romania

Demographic characteristics	
Age	20–29 (*n* = 5) 30–44 (*n* = 9) 45–59 (*n* = 1) Missing data (*n* = 2)
Self-identified gender	Female (*n* = 13) Intersex (*n* = 2) Missing data (*n* = 2)
Country of birth	Poland (*n* = 5) Romania (*n* = 7) Latvia (*n* = 5)
Religion	Christian (*n* = 9) No religion (*n* = 5) Missing data (*n* = 3)
Languages spoken	Polish, Latvian, Romanian, English, Russian, Spanish, French, Hungarian, Italian
Length of residency in the UK	Between 3 and 17 years

#### Clinical research staff

Twenty-two clinical research staff participated across 5 focus groups. Of the clinical staff participants, four self-identified as being born in an EU8/2 country. A wide range of speciality areas were represented, including haematology (*n* = 8), gynaecology and obstetrics (*n* = 4), rheumatology (*n* = 3), oncology (*n* = 3), cardiology (*n* = 2), renal (*n* = 2), otology (*n* = 1), pharmacy (*n* = 2), ophthalmology and clinical trials (*n* = 2) and paediatrics (*n* = 1).

### Template analysis

**Fig. 2:**
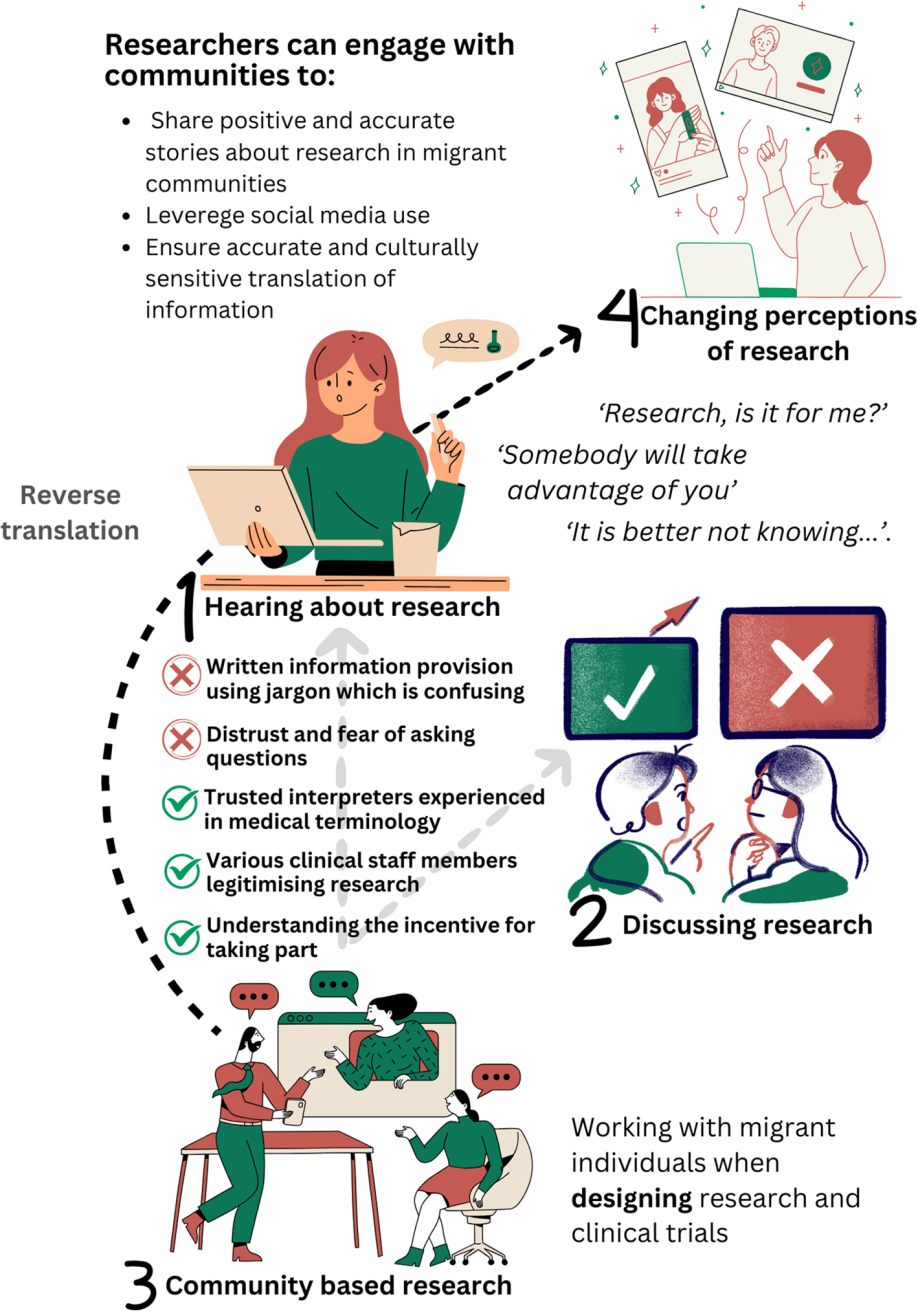
A summary of research findings outlining processes of how migrant individuals in this study respond when hearing about research (1); their experiences of discussing research (2) and how a community approach to research can facilitate recruitment (3) through reverse translation (4) of knowledge

Iterative discussions between NM and BC led to the refinement of the 4-theme template into a 3-theme structure: (1)Aambivalence, misunderstanding and fear shape cancer research perceptions; (2) Structural barriers and gaps in cultural competency; and (3) Building trust through community engaged research. Interpretations are evidenced using participant quotations taken directly from transcripts. Where possible we also indicate whether a finding was endorsed by one individual or by many focus group participants. All themes have been written to capture the perspectives of migrant individuals (labelled as Latvian FG, Romanian FG or Polish FG) and clinical research staff labelled per focus group as CRSW 1, 2, 3, 4 or 5. A summary of the study results (from both migrant participants and clinical research staff) is illustrated in Fig. 2.

### Theme 1: Ambivalence, misunderstanding and fear shape cancer research perceptions

Migrant individuals and clinical staff highlighted that perceptions of cancer research may have underlying influences on people’s willingness and trust to participate in related trials. Such perceptions could be understood as feelings of ambivalence towards research (i.e. having both positive and negative views of research) alongside potential misunderstandings on the reasons for and consequences of doing research. Additionally, cancer itself was associated by some with fear and a lack of hope; however, there were stories and suggestions which may have implications and pose opportunities for cancer research participation.

#### A lack of familiarity with research practices

Perspectives of migrant individuals towards cancer research varied with some participants echoing their general support for research: ‘*I want to put a big, bold dot here and say this about research. I’m very positive about the research you’re doing, and I do appreciate researchers*’ (Latvian FG)*.* At the same time, they acknowledged a gap with regard to research participation and healthcare utilisation on behalf of their communities. For some, a lack of understanding and familiarity with research processes was highlighted: *They [referring to Polish community] think – this is a nuisance. Or maybe they lack understanding, awareness about the importance of research and how they input can help, how they views can help *in *developing new treatments, medications'* (Polish FG), illustrating how people’s perceptions of the inconvenience of research participation in combination with a lack of awareness of its value can produce participation barriers, whilst increasing such awareness could act as a potential opportunity: ‘*People need to be encouraged and informed that participating in research is important, not just for themselves, but for future generations*’ (Latvian FG). In many cases, a lack of familiarity with research was associated with mistrust and fear: *There is a limited understanding of research, and then because you do not know, it feels scary* (Romanian FG)*.* Accounts from clinical research staff also spoke to this fear and mistrust in relation to its origins in historical research legacies which are embedded in unethical and disempowering processes: ‘*They don’t see the research as improvement of medicine in general it’s just manipulation of certain [good] and it’s not necessarily for your benefit it’s just somebody else will take advantage of you that’s how its seen*’ (CRSW 4).

Participants also highlighted how they rarely came into contact with research, which can lead to a lack of identification with research practices: '*Maybe now it’s different, but we don’t have massive experience to take part in researches most of the time we do not receive letters at home saying- take part in research'* (Latvian FG). In the absence of individual targeted recruitment strategies, this indicates the importance of increasing trustworthy dialogue and discourse around research in community and online spaces wherein potential participants may engage with research-related recruitment and content.

#### Cancer fear may hinder participation

In the case of cancer research specifically, attitudes of participants towards research participation were often driven by fear. Commonly referenced perceptions of ‘cancer research’ included receiving cancer treatment, that research practices could be painful, and in some cases, that participating could lead to getting the disease or being diagnosed with cancer: ‘*I think one of the reasons for not coming forward for research is a fear. Fear of knowing or being diagnosed with something. Sometimes is better not knowing. People are scared to be part of things*’ (Polish FG). It was also evident that the topic of cancer was seen as being fatalistic amongst some participants when speaking on behalf of their communities: ‘*The C word -it’s frightening. It is something unknown. For them [Romanian communities] if they hear the word cancer, that is the most terrible thing*’ (Romanian FG). For some, this associated fear had culminated in a lack of dialogue around cancer for them personally: ‘*For me, it’s something very scary. Something I don’t want to think about something about. I don’t want to talk*’ (Latvian FG). Migrant participants explained that negative portrayals of cancer experiences were often maintained through sharing stories of death and hopelessness, as illustrated by a Romanian participant:

I just want to give my opinion related to your last question why people probably do not want to take part in research. Maybe because we did not hear a lot of positive feedback. I mean all we hear around us is that that person has got cancer. Sometimes we don’t know the end of the story if that person made it or survive, or if they struggle. Uh, we just hear the bad news that a person got cancer and that is it. (Romanian FG)

On the other hand, stories of hope from participants’ communities were also referred to, and these were mostly associated with religiosity and faith: '*There are people in the community highly religious and they are absolutely sure that God is going to cure them'* (Romanian FG). Despite reference to stigma and taboo associated with having cancer, it was suggested that it is becoming more commonplace to be open and speak about the disease: '*Yes, people talk more openly about having cancer: Before when you were diagnosed, the doctors didn’t tell you that you had cancer, your family kept it hidden from you'* (Latvian Polish FG). Thus, misperceptions of cancer research such that it can promote cancer, alongside deeply rooted fears and taboo around cancer can create apprehension around trial participation which represent important barriers to research engagement. Yet, potential increases in the awareness of rising cancer incidences may present an opportunity for clinical research engagement.

### Theme 2: Structural barriers and gaps in cultural competency

Throughout the clinical staff focus groups, it was highlighted that there is a gap in participants’ hospital recruitment settings between the cultural competency values needed to design and enact inclusive research practices and the structural opportunities for this.

Clinical research staff, some of whom had migrated from EU8/2 countries themselves, acknowledged that engaging with migrant individuals from EU8/2 countries may require specific cultural competency skills. For example, they reflected on the role of culture and research education, and assumptions related to research participation that migrant individuals might hold. Practical considerations such as language and time pressures were also salient considerations relating to research, along with communication styles, language and time pressures on potential participants:

Not only do we have a language barrier, it’s very often not in their own language which makes it even more difficult to spend a bit more time, and the patient probably hasn’t got enough time to spend on the research discussion because they can’t have time off work and family issues as well and so there just isn’t enough time for the discussion. (CRSW 5)

Despite noting how different engagement strategies may be needed depending on patients’ ethnic backgrounds, it seemed that the structure of clinical research practices often did not allow for values of cultural competency to be meaningfully implemented. Examples of such discrepancies included the nine to five working day structure of clinical research staff, a lack of proficient translation services and a general lack of time to explain research: ‘*We got our time issues as well so we don’t have enough time to spend with a patient to assist with language barriers, and that may depend on whether we have a translator*’ (CRSW 5). Thus, these embedded structures do not take into consideration the flexibility needed to endorse inclusive and equitable engagement practices. For example, clinical staff suggested that for some migrant individuals, it may be important to engage with their family members to build trust and to aid translation; however, caution with this approach was advised and the need for an independent interpreter to explain the study requirements was emphasised: '*So what we are not allowed to do is of course use a relative as an interpreter'* (CRSW 4).

The need to consider the caring responsibilities of children or other family members and how patients can be best supported and incentivised to participate in research studies was also felt to be important by staff given the typically younger demographic of the migrant population, although the ethical implications of incentivising research participation were raised as a concern: ‘*We know that by saying that [incentive] we will get recruits but are we allowed to say it because it should be voluntary? It puts the researcher in a difficult position*’ (CRSW 1). Thus, navigating conversations to ensure equal consideration of the benefits and risks of a study was viewed to be paramount, enabling participants to give truly informed consent if they decided to participate. Therefore, whilst flexibility in approaching patients could benefit and promote effective engagement, there are often ethical and structural barriers in place which prohibit such cultural competency to be acted upon.

### Theme 3: Building trust through community engaged research

Both migrant individuals and clinical research staff suggested solutions for increasing engagement which were embedded in participatory approaches and forms of community engagement. Researching *with* rather than *on* migrant individuals through co-creation and co-design approaches can stimulate trust through refining traditional research practices and can be pivotal in legitimising the prospect of cancer research participation.

#### Co-researching with communities

It was strongly emphasised that researchers needed to liaise with clinicians and members of the participant community when conceptualising and designing research to stimulate cultural understanding and tailoring of research, rather than this being an afterthought at the stage of recruitment:

When they [researchers] formulate and specify the research question, that should inform the design. You have to ask yourself what you want to achieve, if you want to get an Eastern European person on board you know how you will facilitate that, something that person can feel attracted to. (CRSW 4)

An emphasis on clarity with regard to the intentions and aims of research was further highlighted by migrant individuals as having consequences for their understanding of research:

I think some people may have different understanding on what research is. When you say about being part of cancer research, are you talking about trials, where the patients are having new medicine tested or are you talking about general research where people just say share their experiences. Sometimes this is not explained well. (Romanian FG)

Examples of taking a community approach early on in the research process included going into public spaces and engaging with community champions who could act to build and foster trust between the research team and communities, alongside aiding the translation of the meanings of research to avoid the development of misconceptions: ‘*There are multiple interpretations, and when people aren’t directly involved in research projects, the term “research” itself can be quite unclear*’ (Latvian FG). Migrant individuals also highlighted the effectiveness of sharing positive narratives around cancer research that challenge dominant negative thinking around participation as a potentially successful means of outreach (Latvian FG). This was echoed by others who highlighted the importance of building trust in their communities through sharing stories with other women: '*We share and talk between us about the experience, because your experience will actually make a mark on you, on your trust'* (Romanian FG). Thus, engaging with the target population at an early stage in order to design research so that it motivates potential participants on their terms underscored an equitable approach to engagement from the perspective of citizens and staff.

#### Incentivising and legitimising research

Avenues for making research participation more attractive to migrant individuals, alongside legitimising the status of research studies, were highlighted as strategies for increasing engagement. Clinical research staff advanced the importance of incentives for research participation, particularly for studies where participants themselves may not benefit directly. Participants highlighted the importance of supplementary benefits in encouraging participation, enhancing the overall research experience and acting as a motivator (Polish FG). It was noted that offering additional advantages such as free health assessments or diagnostic tests, alongside study participation, can significantly improve the attractiveness of the research: '*Having discounts for health checks, free breast screening- this would be great. Something for me, health benefits' *(Polish FG). This was further supported by a Polish clinical staff member who reflected on the limits of monetary compensation: '*In Poland, from my perspective, twenty pounds is not that big, so they will probably be more happy with an extra scan. It’s nice if they can have their parking covered actually, they will be more keen'* (CRSW 1).

Given that it is common for the legitimacy of research to be questioned and scrutinised, clinical staff suggested that buy-in from clinical teams at various levels was an essential avenue to support research and reassure potential participants of its safety. In particular, endorsement of research from clinicians at various structural levels was highlighted: '*I think we have got a massive push to try and engage a lot of our clinical staff so that it’s seen as everybody’s role so that when patients are touching the wards or in clinic that research is something that is discussed'* (CRSW 2). Thus, wider engagement may also lead to potential participants being more likely to hear about research. Alongside this, many clinical research staff suggested that the role of the doctor was important in the legitimisation process, for example *If it comes from the doctor they will consider it, that is important definitely in Polish community, but let’s say my colleague will come and talk to them, that will not be hard'* (CRSW 1). Migrant individuals frequently referred to how their communities often distrusted the intentions of doctors, and instead, it was suggested that legitimisation be enhanced through making the aims and motivations of the research clear and accessible to potential participants.

## Discussion

This study highlights key considerations when aiming to recruit migrant individuals from EU8/2 countries into clinical cancer research trials. The findings represent a complex interplay of individual- and group-level factors alongside structural and institutional barriers. Such considerations can be broadly divided into factors related to changing perceptions of individuals regarding research and normalising participation; considerations when discussing and explaining research studies to migrant individuals from a logistical and clinical perspective; and how research design needs to be shifted to accommodate the needs of migrant individuals (Fig. 2).

Migrant individuals highlighted the negative perceptions and defensive attitudes that their communities hold regarding clinical cancer research participation. Such perceptions were predominantly shaped by a lack of familiarity with research practices. Whilst a lack of trust in clinical staff and healthcare-related institutions was salient and was thought to be related to historical legacies of research cultures in participants’ countries of birth, factors related to a strong fear of cancer and medical discovery also added complexity to this understanding. Distrust of research and researchers is common amongst ethnic minority groups [22] and has been linked to a lack of knowledge and understanding of research itself [23]. However, recently, scholars have prompted a reframing of this phenomenon to consider more overtly the *trustworthiness* of researchers and the efforts that are made to enable inclusive engagement rather than maintaining a focus on community distrust [24]. Shifting focus to how research can be presented to communicate its trustworthiness was also evident in this study with participants calling for clearer and more accessible research proposals. Increased trustworthiness via sharing positive and accurate stories from relatable individuals who have previously participated in research or from peer researchers and community champions [25] was also suggested.

At the point of considering whether to participate in a research trial, we found that language and associated communication strategies alongside building trust were significant and enmeshed considerations. Language barriers, particularly for those who are not proficient in English, are frequently cited with reference to limiting one’s understanding of research [26], one’s ability to ask questions about research, and to developing trusting relationships with research staff [27]. Research staff play a pivotal role in facilitating communication and building trust with participants. However, research staff may face challenges in assessing participants’ comprehension, addressing questions and ensuring adequate language support, particularly in the context of adverse events. Thus, considerations about the validity of consent and participants’ ability to adhere to study protocols were key concerns related to English literacy levels.

In complex trials, where accurate translation and interpretation are critical to ensuring valid consent, trusted interpreters with expertise in medical terminology could help to bridge these communication gaps effectively. Thus, the inclusion of culturally competent staff, particularly those from ethnic minority groups who possess additional language skills [28], alongside the engagement of a diverse range of clinical staff (including doctors and allied healthcare professionals) can help address these challenges. Such staff members can bridge cultural and linguistic divides, fostering trust and rapport with participants whilst enhancing the inclusivity and effectiveness of the research process.

Lastly, an important finding from exploring experiences of migrant individuals and staff was a disconnect between the aims of research studies and the potential for these to be met in current clinical recruitment practices. This disconnect was thought to contribute to the development of mistrust between individuals and researchers, and to inefficient research practices. Taking a community-oriented approach which genuinely and transparently engages with individuals during design phases of research was suggested to be a key avenue to connect underserved communities with health research. Examples of engagement included consulting with communities when designing research questions and prioritising research agendas. They also involved considering how to communicate research effectively (e.g. through video, audio, written or visual means) and leveraging mass media platforms. Additional strategies included ensuring that translated materials retained their original meaning and nurturing relationships with community leaders who acted as trusted links between health research teams and underserved communities. Such initiatives not only raise awareness but also build trust and promote a culture of research engagement within communities [29–31]. Building relationships between communities and research teams also enables the process of reverse translation, whereby knowledge derived from communities can be effectively mobilised to inform research practices, and vice versa [32]. Moreover, culturally competent research teams are better equipped to understand the unique needs and concerns of diverse participant groups.

### Strengths and limitations

To our knowledge, this is the first study to elevate the experiences and views of individuals who have migrated to the UK from an EU8/2 country. A key strength of this research is its multidisciplinary and culturally diverse sample and its approach of exploring experiences from both participants and clinical research staff to identify mechanisms underpinning experience and potential intervention areas. The choice to use online focus groups to explore migrant individuals’ experiences related to barriers and opportunities of research participation was informed by the needs of the research question, participant preference, and financial and time considerations of including an interpreter. Despite this, we acknowledge that such an approach perhaps did not provide space for nuanced and/or outlier individual experiences to be explored in depth. This approach is further limited in that it may have facilitated the homogenisation of culturally or ethnically generalised experiences, as we saw that some participants tended to speak generally about the communities they identify with rather than their own personal experience. Thus, whilst the study aimed to represent the experiences of migrant individuals from EU8/2 countries and whilst these results provide indication of barriers and opportunities from the reported experiences related to migrants, these findings cannot and should not be generalised to broader cultural and/or ethnic communities as a whole.

### Research dissemination

In order to mobilise the knowledge generated from this research, an infographic for migrant individuals was designed and created with the aim of providing information on the meanings and practices of cancer research. Data from the three focus groups was used to design an initial English language infographic with topics covering ‘*what is cancer research?*’; ‘*why take part in cancer research?*’; ‘*how many people participate in cancer research every year?*’; and ‘*questions to ask before deciding to participate in research*’. Those who took part in the focus groups were invited to review the infographic before it was translated into Romanian, Polish and Latvian (Supplementary data). The final translations were then checked by native Romanian, Latvian and Polish speakers who worked in a clinical capacity to ensure comprehension and accuracy.

## Conclusions

Enhancing the trustworthiness of clinical cancer research at various stages of the engagement process (i.e. when designing, communicating and recruiting) is necessary in order to move towards inclusive research engagement. Participant-facing research materials which do not consider the intersectionality characteristics of potential participants (e.g. not being proficient in the English language) create a variety of recruitment barriers pertaining to a lack of understanding of research and questions around valid consent alongside. Engaging with members of the target community during design phases can enable relevant considerations of incentivising and legitimising research, along with inclusive modes of information provision and outreach.

## Supplementary Information

Supplementary Material 1.

## Data Availability

The participants of this study did not give written consent for their data to be shared publicly, so due to the sensitive nature of the research supporting data is not available.

## References

[1] Oyer RA, et al. Increasing racial and ethnic diversity in cancer clinical trials: an American Society of Clinical Oncology and Association of Community Cancer Centers joint research statement. J Clin Oncol. 2022;40(19):2163–71. 35588469 10.1200/JCO.22.00754

[2] Helm A, et al. Strategies for recruiting participants underrepresented in clinical research: a scoping review. Soc Sci Med. 2025;385:118603. 41005122 10.1016/j.socscimed.2025.118603 PMC12628295

[3] Health Research Authority, N.H.S.. Increasing the diversity of people taking part in research. 2024; Available from: https://www.hra.nhs.uk/planning-and-improving-research/best-practice/increasing-diversity-people-taking-part-research/.

[4] Murali M, et al. Ethnic minority representation in UK COVID-19 trials: systematic review and meta-analysis. BMC Med. 2023;21(1):111. 36978166 10.1186/s12916-023-02809-7 PMC10049782

[5] Guerrero S, et al. Analysis of racial/ethnic representation in select basic and applied cancer research studies. Sci Rep. 2018;8(1):13978. 30228363 10.1038/s41598-018-32264-x PMC6143551

[6] Bodicoat DH, et al. Promoting inclusion in clinical trials-a rapid review of the literature and recommendations for action. Trials. 2021;22(1):880. 34863265 10.1186/s13063-021-05849-7 PMC8643184

[7] Skea ZC, Treweek S, Gillies K. It’s trying to manage the work’: a qualitative evaluation of recruitment processes within a UK multicentre trial. BMJ Open. 2017;7(8):e016475. 28801422 10.1136/bmjopen-2017-016475 PMC5629709

[8] Zucchelli F, et al. Recruiting to cohort studies in specialist healthcare services: lessons learned from clinical research nurses in UK cleft services. J Clin Nurs. 2018;27(5–6):e787-97. 29193429 10.1111/jocn.14188

[9] Rodríguez-Torres E, González-Pérez MM, Díaz-Pérez C. Barriers and facilitators to the participation of subjects in clinical trials: an overview of reviews. Contemp Clin Trials Commun. 2021;23:100829. 34401599 10.1016/j.conctc.2021.100829 PMC8358641

[10] Pothuri B, et al. Inclusion, diversity, equity, and access (IDEA) in gynecologic cancer clinical trials: a joint statement from GOG foundation and Society of Gynecologic Oncology (SGO). Gynecol Oncol. 2023;174:278–87. 37315373 10.1016/j.ygyno.2023.05.006

[11] Mandane B, et al. Attitudes and barriers to participation in window-of-opportunity trials reported by White and Asian/Asian British ethnicity patients who have undergone treatment for endometrial cancer. Trials. 2023;24(1):754. 38007461 10.1186/s13063-023-07572-x PMC10676569

[12] GOV.UK. Healthcare for EU citizens living in or moving to the UK. 2024; Available from: https://www.gov.uk/guidance/healthcare-for-eu-and-efta-nationals-living-in-the-uk.

[13] Gallup. Wellcome Global Monitor. First wave findings. 2019.

[14] Office for National Statistics. Population of the UK by country of birth and nationality: 2020. 2021; Available from: https://www.ons.gov.uk/peoplepopulationandcommunity/populationandmigration/internationalmigration/bulletins/ukpopulationbycountryofbirthandnationality/2020.

[15] Patel H, et al. Awareness of and attitudes towards cervical cancer prevention among migrant Eastern European women in England. J Med Screen. 2020;27(1):40–7. 31514572 10.1177/0969141319869957

[16] Witham MD, et al. Developing a roadmap to improve trial delivery for under-served groups: results from a UK multi-stakeholder process. Trials. 2020;21(1):694. 32738919 10.1186/s13063-020-04613-7 PMC7395975

[17] Lidvig K, Hillersdal L, Earle D. Interdisciplinary tool helps fast-track interdisciplinary learning and collaboration. Integrative Pathway. 2017;39(2):3–6.

[18] King N. Doing template analysis. Qualitative organizational research: core methods and current challenges. 2012;26:426.

[19] Braun V, Clarke V. Toward good practice in thematic analysis: avoiding common problems and be(com)ing a knowing researcher. Int J Transgend Health. 2023;24(1):1–6. 36713144 10.1080/26895269.2022.2129597 PMC9879167

[20] Office for National Statistics. Language, England and Wales: census 2021. 2021; Available from: https://www.ons.gov.uk/peoplepopulationandcommunity/culturalidentity/language/bulletins/languageenglandandwales/census2021.

[21] The Migration Observatory. Who counts as a migrant? Definitions and their consequences. 2024; Available from: The Migration Observatory (2024, February 22). Who counts as a migrant? Definitions and their consequences. Retrieved from https://migrationobservatory.ox.ac.uk/resources/briefings/who-counts-as-a-migrant-definitions-and-their-consequences/.

[22] Sheridan R, et al. Why do patients take part in research? An overview of systematic reviews of psychosocial barriers and facilitators. Trials. 2020;21(1):259. 32164790 10.1186/s13063-020-4197-3 PMC7069042

[23] Limkakeng A, et al. Willingness to participate in clinical trials among patients of Chinese heritage: a meta-synthesis. PLoS One. 2013;8(1):e51328. 23349672 10.1371/journal.pone.0051328 PMC3547937

[24] Passmore SR, et al. My blood, you know, my biology being out there…”: consent and participant control of biological samples. J Empir Res Hum Res Ethics. 2024;19(1–2):3–15. 38192107 10.1177/15562646231222665 PMC10957312

[25] Quay TA, et al. Barriers and facilitators to recruitment of South Asians to health research: a scoping review. BMJ Open. 2017;7(5):e014889. 28576896 10.1136/bmjopen-2016-014889 PMC5541387

[26] Manthorpe J, et al. We are not blaming anyone, but if we don’t know about amenities, we cannot seek them out’: black and minority older people’s views on the quality of local health and personal social services in England. Ageing Soc. 2009;29(1):93–113.

[27] Sime D. ‘I think that Polish doctors are better’: newly arrived migrant children and their parents’ experiences and views of health services in Scotland. Health Place. 2014;30:86–93. 25237717 10.1016/j.healthplace.2014.08.006

[28] Son J. Assimilation and health service utilization of Korean immigrant women. Qual Health Res. 2013;23(11):1528–40. 24108090 10.1177/1049732313507142

[29] Hussain-Gambles M, Atkin K, Leese B. Why ethnic minority groups are under-represented in clinical trials: a review of the literature. Health Soc Care Community. 2004;12(5):382–8. 15373816 10.1111/j.1365-2524.2004.00507.x

[30] Stepanova V, Poppleton A, Ponsford R. Central and Eastern European migrants in the United Kingdom: a scoping review of the reasons for utilisation of transnational healthcare. Health Expect. 2024;27(4):e14155. 39044675 10.1111/hex.14155 PMC11266902

[31] Santucci C, et al. Persisting cancer mortality gap between western and eastern Europe. Eur J Cancer. 2022;165:1–12. 35189536 10.1016/j.ejca.2022.01.007

[32] Blumenthal D. Reverse translation in health policy and management: from bedside to bench and beyond. Health Serv Res. 2005;40(1):9–18. 15663699 10.1111/j.1475-6773.2005.00339.x PMC1361123

