# Supplementary material for: Barriers to clinical cancer research participation: moving from inclusion to engagement when considering European migrants’ recruitment
Source: Trials. 2025 Dec 23;26:576. doi: 10.1186/s13063-025-09293-9 (PMC12723834; doi:10.1186/s13063-025-09293-9)
Supplement: Supplementary file 1 — Supplementary Material 1. [file 13063_2025_9293_MOESM1_ESM.pdf]

Cancer research studies

What are they and how can you get involved?

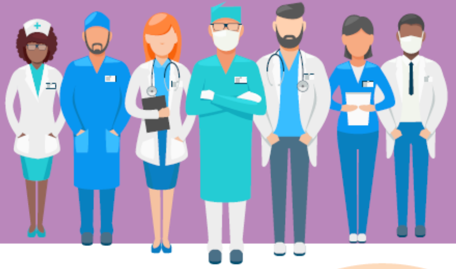

What is cancer research?

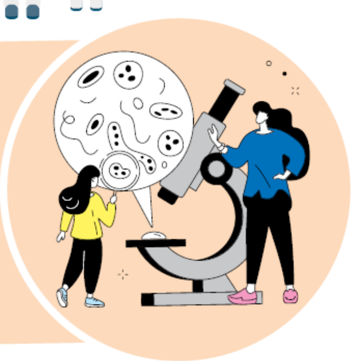

A process that helps us to understand what can cause cancer, stop cancer developing and how cancer can be treated.

Can include a number of activities from telling a researcher your views of cancer to taking part in lab-based studies.

Considers your safety and your wellbeing as its main priority

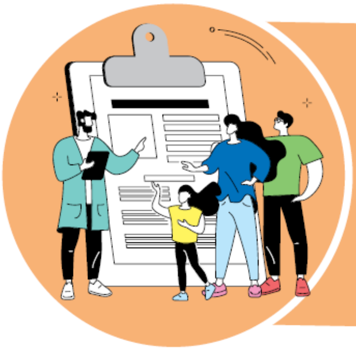

Why take part in research?

To share your views about cancer care

To be more aware about your health

To help others in your communities in the future

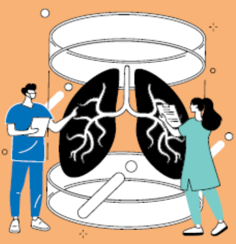

Almost 1 million people in England take part in research every year*

* https://rb.gy/m3fvf

Questions to ask before you decide to take part in research

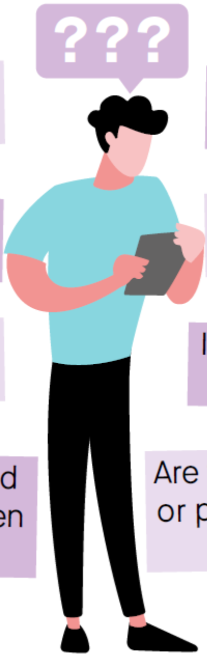

- What does the study involve?
- What is the time commitment?
- Is the research accessible to me?
- Have my cultural and religious values been considered?
- What support is available?
- Is there language support?
- Is it possible to do the study online?
- Are there any physical or psychological risks to taking part?

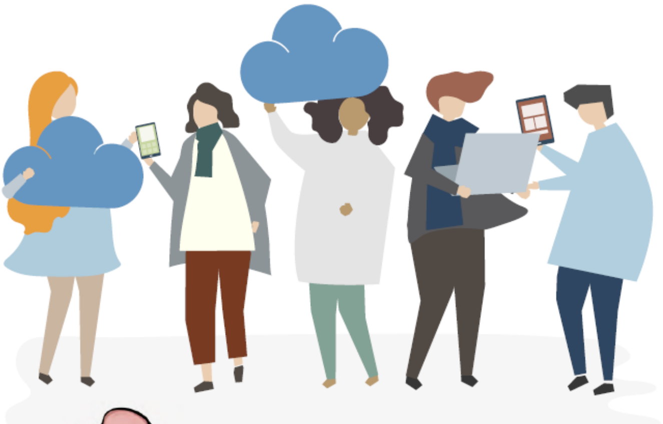

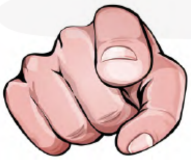

WE NEED YOU TO GET INVOLVED

Please contact your local research team by emailing

calling

or scanning the QR code

UNIVERSITY OF LEICESTER

The Centre for Ethnic Health Research
national centre for tackling health inequalities

NIHR | Clinical Research Network East Midlands

Illustrations by pch.vector / Freepik

Badania naukowe nowotworów

Czym są i jak można się w nie zaangażować?

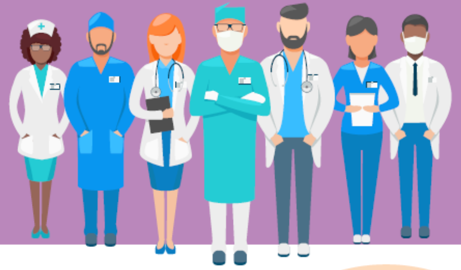

Czym są badania nad nowotworami?

Proces, który pomaga nam zrozumieć, co może powodować i powstrzymywać nowotwory oraz jak można je leczyć

Może obejmować szereg działań, od poinformowania naukowca o swoich poglądach na temat nowotworów, po udział w badaniach

Twoje bezpieczeństwo i dobrostan są głównym priorytetem

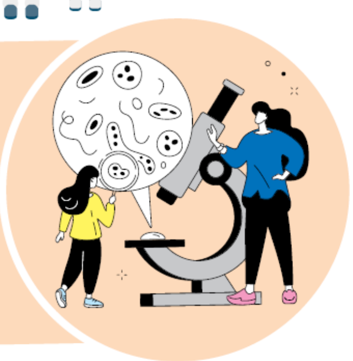

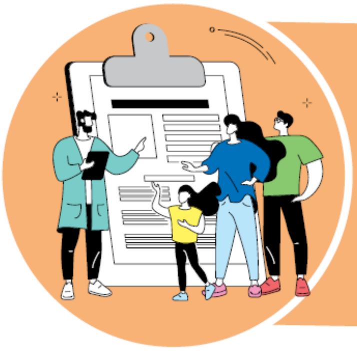

Dlaczego warto brać udział w badaniach?

Aby podzielić się swoimi poglądami na temat opieki nowotworowej

Aby uzyskać więcej informacji o swoim zdrowiu

Aby pomóc innym w Twojej w społeczności

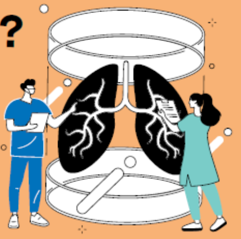

Co roku w badaniach bierze udział prawie 1 milion osób w Anglii

* https://rb.gy/m3fvf

Pytania, które należy zadać przed podjęciem decyzji o wzięciu udziału w badaniach

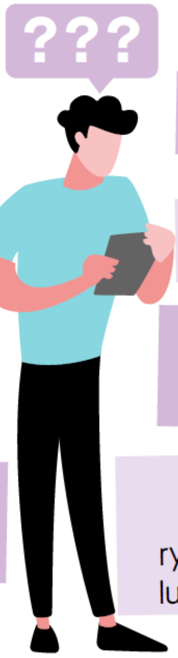

- Co obejmują badania?
- Jakie jest wymagane zaangażowanie czasowe?
- Czy badania są dla mnie przystępne?
- Czy wzięto pod uwagę moje wartości kulturowe i religijne?
- Jakie wsparcie jest dostępne?
- Czy jest wsparcie językowe?
- Czy możliwe jest wzięcie udziału przez Internet?
- Czy uczestnictwo wiąże się z jakimś ryzykiem fizycznym lub emocjonalnym?

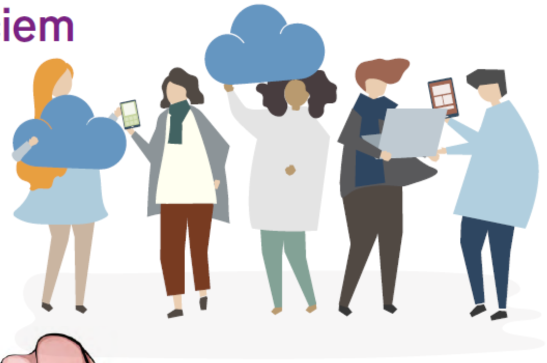

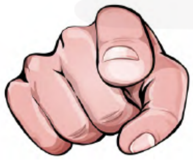

KOLEJNE KROKI

Jeśli chcesz wziąć udział w badaniach w swojej okolicy, skontaktuj się z naszym zespołem na the University of Leicester. Wyślij e-mailem lub zeskanuj poniższy kod QR

UNIVERSITY OF LEICESTER

The Centre for Ethnic Health Research
national centre for tackling health inequalities

NIHR | Clinical Research Network East Midlands

Illustrations by pch.vector / Freepik

Pētījumi vēža izpētei

Kas tie ir, un kā varat iesaistīties?

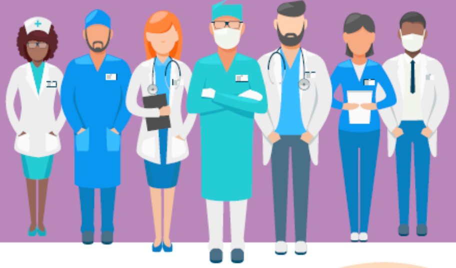

Kas ir vēža izpēte?

Process, kas palīdz mums saprast, kas var izraisīt vēzi, apturēt vēža attīstību un kā var ārstēt vēzi.

Tas var ietvert vairākas aktivitātes — no pētnieka informēšanas par jūsu domām attiecībā uz vēzi līdz dalībai laboratorijās veiktajos pētījumos.

Tajā tiek ņemta vērā jūsu drošība un labsajūta kā galvenā prioritāte.

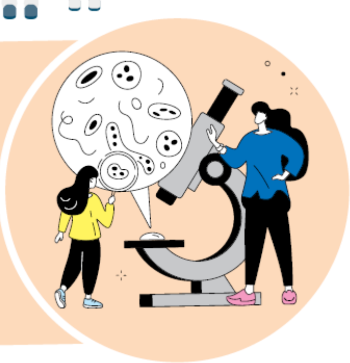

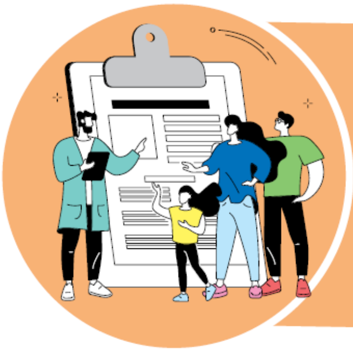

Kāpēc piedalīties izpētē?

Lai dalītos savās domās par vēža slimnieku aprūpi

Lai labāk pārzinātu savu veselību

Lai nākotnē palīdzētu citām personām jūsu kopienās

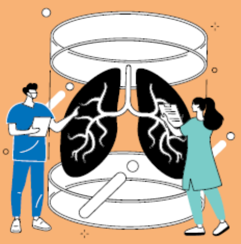

Apvienotaja Karalistē gandrīz 1 miljons cilvēku katru gadu piedalās zinātniskajos pētījumos https://rb.gy/m3fvf

Vai jums ir kādi jautājumi, kurus vēlaties uzdot, pirms izlemt par dalību izpētē?

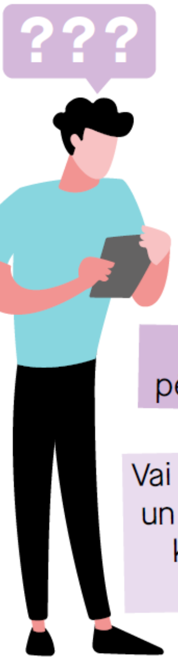

Ko ietver pētījums?

Cik daudz laika prasīs dalība pētījumā?

Vai izpēte man ir pieejama?

Vai ir ņemtas vērā manas kultūras un reliģiskās vērtības?

Kāds atbalsts ir pieejams?

Vai ir pieejams valodas atbalsts?

Vai ir piedalīties pētījumā tiešsaistē?

Vai pastāv kādi fiziskie un psiholoģiskie riski, kas saistīti ar dalību pētījumā?

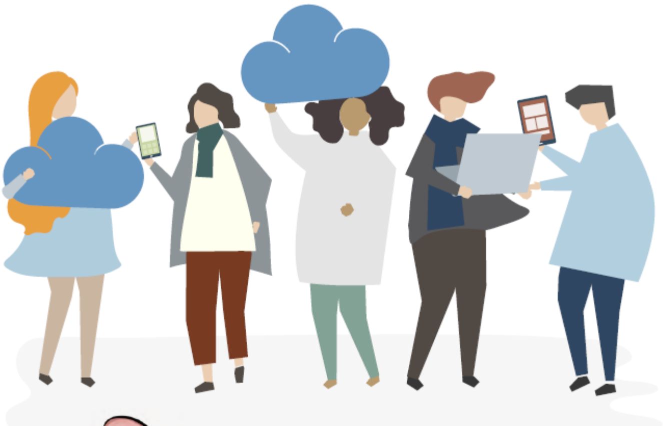

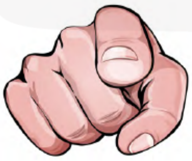

Jūs esat mums nepieciešami — iesaistieties!

Lūdzu, sazinieties ar vietējo izpētes komandu, sūtot ziņojumu uz e-pasta adresi

zvanot pa tālrun

vai noskenējot QR kodu

University of Leicester

The Centre for Ethnic Health Research
national centre for tackling health inequalities

NIHR | Clinical Research Network East Midlands

Illustrations by pch.vector / Freepik

Studii clinice oncologice

Ce sunt aceste studii și cum puteți participa?

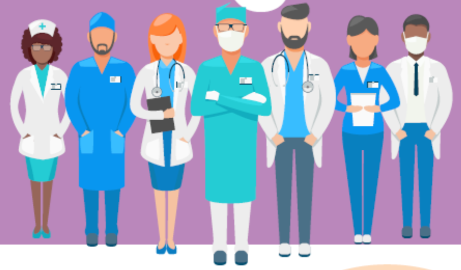

Ce este un studiu clinic oncologic?

Un proces care ne ajută să înțelegem ce poate cauza cancer, cum se oprește evoluția cancerului și cum poate fi tratat cancerul.

Poate include mai multe activități, de la comunicarea părerii dumneavoastră despre cancer cercetătorului din cadrul studiului clinic, până la participarea la studii de laborator.

Consideră prioritare siguranța și bunăstarea dumneavoastră.

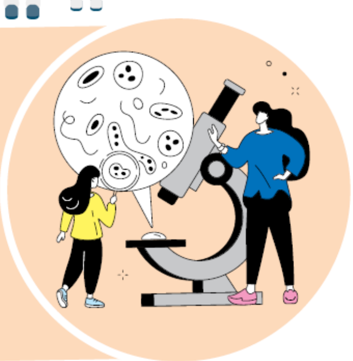

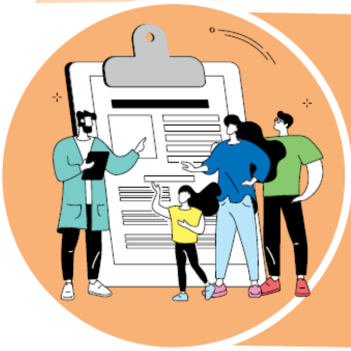

De ce să particip la studiu?

Pentru a vă spune părerea despre asistența medicală acordată persoanelor cu cancer.

Pentru a conștientiza mai mult implicațiile pentru sănătatea dumneavoastră.

Pentru a-i ajuta pe viitor pe cei din comunitățile dumneavoastră.

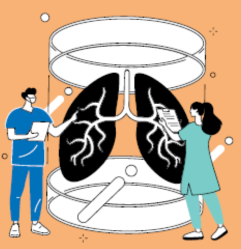

Approximativ 1 milion de persoane în Anglia participă în studii de cercetare anual https://rb.gy/m3fvf

Întrebări pe care le puteți adresa înainte de a decide să participați la studiu:

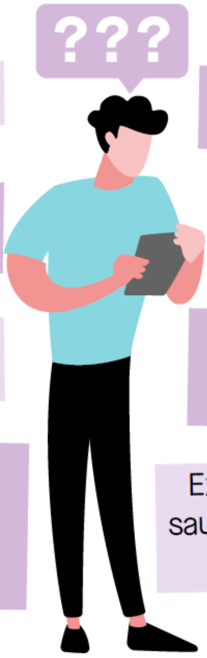

- Ce implică studiul?
- Ce sprjin pot primi în cadrul studiului?
- Cât timp necesită participarea mea?
- Pot beneficia de traducere?
- Am acces la cercetare?
- Pot participa online la acest studiu?
- S-au luat în considerare valorile mele culturale și religioase?
- Există vreun risc fizic sau psihologic asociat participării mele?

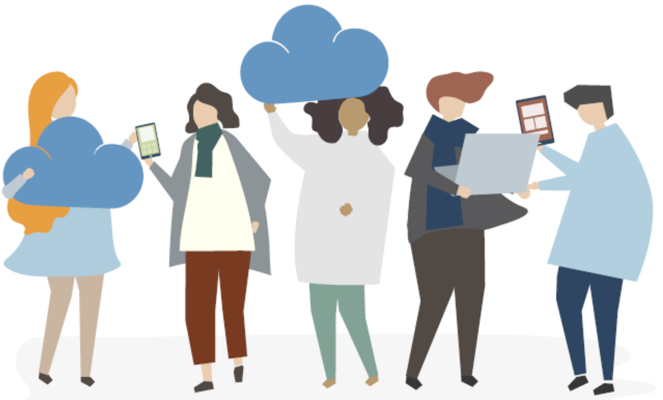

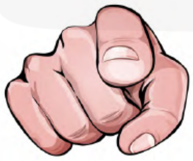

Avem nevoie de implicarea dumneavoastră

Vă rugăm să contactați echipa de cercetare la adresa de e-mail
la numărul de telefon sau prin scanarea codului QR.

University of Leicester

The Centre for Ethnic Health Research
national centre for tackling health inequalities

NIHR | Clinical Research Network East Midlands

Illustrations by pch.vector / Freepik
